# MULTIPLE CHRONIC CONDITIONS IN OLDER CANCER SURVIVORS WITH AND WITHOUT HIV

**DOI:** 10.1093/geroni/igz038.1158

**Published:** 2019-11-08

**Authors:** Siran Koroukian, Nicholas Schiltz, Johnie Rose, Gregory Cooper, Cynthia Owusu, Abby Statler, Scott E Moore, Sarah Markt

**Affiliations:** 1 Case Western Reserve University, Cleveland, Ohio, United States; 2 Cleveland Clinic, Cleveland, Ohio, United States; 3 Frances Payne Bolton School of Nursing, Case Western Reserve University, Cleveland, Ohio, United States

## Abstract

Background: With more effective treatment for both HIV and cancer, longevity among persons living with HIV (PLwHIV) has improved significantly. However, little is known about whether the comorbidity profile of cancer survivors differs between PLwHIV and their HIV-free counterparts. To address this critical gap in knowledge, we compared the occurrence and combination of multiple chronic conditions (MCCs) among older cancer survivors by HIV status. Methods: We used national data from the 2014 Chronic Conditions Data Warehouse (CCW) as part of the Medicare Beneficiary Summary File, which flags 66 conditions, including HIV/AIDS, and history of common cancers (colorectal, lung, prostate, and leukemias/lymphomas). We limited our study population to men age 65 years or older who were cancer survivors. In addition to descriptive analysis, we conducted association rule mining (ARM) analysis to compare the prevalence of the most common MCCs among cancer survivors, with and without HIV. Results: We identified 1.3 million individuals, of which 1,901 (0.15%) were PLwHIV. Compared to their HIV-free counterparts, PLwHIV were younger (mean of 72.5 and 77.0 years); more were non-White (41.8% vs. 13.5%); and more of them presented with anemia (44.8% vs. 35.3%), chronic kidney disease (CKD, 41.9% vs. 26.6%), depression (26.6% vs. 13.9%), viral hepatitis (20.4% vs. 0.70%), and/or liver disease (10.7% vs. 4.8%; p < 0.001 for all comparisons). ARM results showed a prominence of CKD in the most common top 5 combinations of conditions. Conclusion: Despite being younger than their HIV-free counterparts, PLwHIV with history of cancer carry a significantly greater comorbidity burden.

